# The Genotype and Phenotype of Proline-Rich Transmembrane Protein 2 Associated Disorders in Chinese Children

**DOI:** 10.3389/fped.2021.676616

**Published:** 2021-05-10

**Authors:** Han-yu Luo, Ling-ling Xie, Si-qi Hong, Xiu-juan Li, Mei Li, Yue Hu, Jian-nan Ma, Peng Wu, Min Zhong, Min Cheng, Ting-song Li, Li Jiang

**Affiliations:** ^1^Department of Neurology, Children's Hospital of Chongqing Medical University, National Clinical Research Center for Child Health and Disorders, Ministry of Education Key Laboratory of Child Development and Disorders, Chongqing, China; ^2^Chongqing Key Laboratory of Pediatrics, Chongqing, China

**Keywords:** PRRT2, genotype, phenotype, benign familial infantile epilepsy, treatment, prognosis

## Abstract

**Objectives:** To study the genetic and clinical characteristics of Chinese children with pathogenic proline-rich transmembrane protein 2 (*PRRT2*) gene-associated disorders.

**Methods:** Targeted next generation sequencing (NGS) was used to identify pathogenic *PRRT2* variations in Chinese children with epilepsy and/or kinesigenic dyskinesia. Patients with confirmed *PRRT2*-associated disorders were monitored and their clinical data were analyzed.

**Results:** Forty-four patients with pathogenic *PRRT2* variants were recruited. Thirty-five of them (79.5%) had heterozygous mutations, including 30 frameshifts, three missenses, one nonsense, and one splice site variant. The c.649dupC was the most common variant (56.8%). Eight patients (18.2%) showed whole gene deletions, and one patient (2.3%) had 16p11.2 microdeletion. Thirty-four cases (97.1%) were inherited and one case (2.9%) was *de novo*. Forty patients were diagnosed with benign familial infantile epilepsy (BFIE), two patients had paroxysmal kinesigenic dyskinesia (PKD) and two had infantile convulsions and choreoathetosis (ICCA). Patients with whole gene deletions had a later remission than patients with heterozygous mutations (13.9 vs. 7.1 months, *P* = 0.001). Forty-two patients were treated with antiseizure medications (ASMs). At last follow-up, 35 patients, including one who did not receive therapy, were asymptomatic, and one patient without ASMs died of status epilepticus at 12 months of age. One patient developed autism, and one patient showed mild developmental delay/intellectual disability.

**Conclusion:** Our data suggested that patients with whole gene deletions could have more severe manifestations in *PRRT2*-associated disorders. Conventional ASMs, especially Oxcarbazepine, showed a good treatment response.

## Introduction

Pathogenic mutations in the proline-rich transmembrane protein 2 (*PRRT2*) gene have recently been identified in a series of disorders. Among these *PRRT2*-associated disorders, a majority of patients have benign familial infantile epilepsy (BFIE), characterized by self-limiting seizures, which usually onset before the age of 6 months, and normal psychomotor development (1), paroxysmal kinesigenic dyskinesia (PKD), characterized by short attacks of dyskinesia triggered by sudden voluntary movements (2), or infantile convulsions with choreoathetosis (ICCA), presenting paroxysmal movement disorders during childhood after remission of infantile convulsions (3). Other rare cases, including hemiplegic migraine and episodic ataxia, have rapidly expanded the boundary of this spectrum (4). Most patients have a tendency to natural remission and favorable outcome. The diverse clinical manifestations caused by variants in the same gene indicate a conventional spectrum that share similar clinical and genetic features, although the pathophysiology of variants in *PRRT2* is not entirely understood.

In this study, we aimed to describe clinical and genetic features of a selected cohort of Chinese children with *PRRT2*-associated disorders and discuss the treatment strategies and prognosis of these patients.

## Materials and Methods

### Patients

A total of 44 children who were diagnosed and treated at the Children's Hospital of Chongqing Medical University were recruited between April 2016 and May 2019. Inclusion criteria for the study were: (I) children (age < 18 years) receiving a new diagnosis of epilepsy or children presenting with paroxysmal movement disorders; (II) patients with pathogenic *PRRT2* variants. Clinical data, including demographic information, family history, clinical manifestation, electroencephalogram (EEG), brain magnetic resonance imaging (MRI), treatment, and follow-up were collected and analyzed. BFIE was defined when patients met the following conditions: (I) onset of seizures between the age of 3 and 12 months; (II) focal seizures with or without secondary generalization; (III) normal interictal EEGs and brain MRI; (IV) normal psychomotor development; (V) positive family history; (VI) seizure-free before 2 years of age; and (VII) exclusion of other metabolic disorders (5). The diagnostic criteria of PKD were revised from Bruno et al. (6) as follows: (I) an identified kinesigenic trigger for attacks; (II) duration of attacks <1 minute; (III) attacks without loss of consciousness or pain; (IV) response to antiseizure medications (ASMs); (V) age of onset 1-20 years old; and (VI) exclusion of other organic diseases. ICCA was diagnosed when patients presented with both BFIE and PKD. All patients participated in a follow up at least 3 months after inclusion in the study, including number of seizure episodes, developmental milestones, mental conditions, and therapeutic regimes.

### Genetic Analysis

Written informed consent was obtained from legal guardians of patient. This study was approved by the Children's Hospital of Chongqing Medical University Ethics Committee. Genomic DNA from each individual and 50 normal controls without epilepsy or any related history were extracted from peripheral blood leukocytes. A custom-designed panel that captured the coding exons of 535 genes associated with epilepsy, including *PRRT2*, was synthetized using the Agilent SureSelect Target Enrichment System which contained a total of 12,000 probes covering 1.285 Mbp. Then, targeted next generation sequencing (NGS) was performed following previously reported experimental procedures (7). Sequencing results were aligned to the Genome Reference Consortium Homo sapiens (human) genome assembly GRCh37 (GRCh37/hg19) and compared with the established human *PRRT2* sequences (NM_145239). Sanger sequencing was performed to validate variants identified by NGS and for segregation analysis. We used PolyPhen-2, SIFT, MutationTaster, and FATHMM-MKL to predict the pathogenicity of the missense variants.

### Statistical Analysis

Continuous variables were expressed as mean ± SD. Differences were tested using Student's *t*-test or the Mann–Whitney *U*-test. Categorical variables were summarized as percentages, and compared byχ^2^ test or by Fisher's exact test. A *P*-value ≤ 0.05 in a two-tailed test was considered statistically significant. Statistical analyses were performed using SPSS version 23.0 (IBM, Armonk, NY).

## Results

### Identification of Pathogenic *PRRT2* Variants

**Figure 1:**
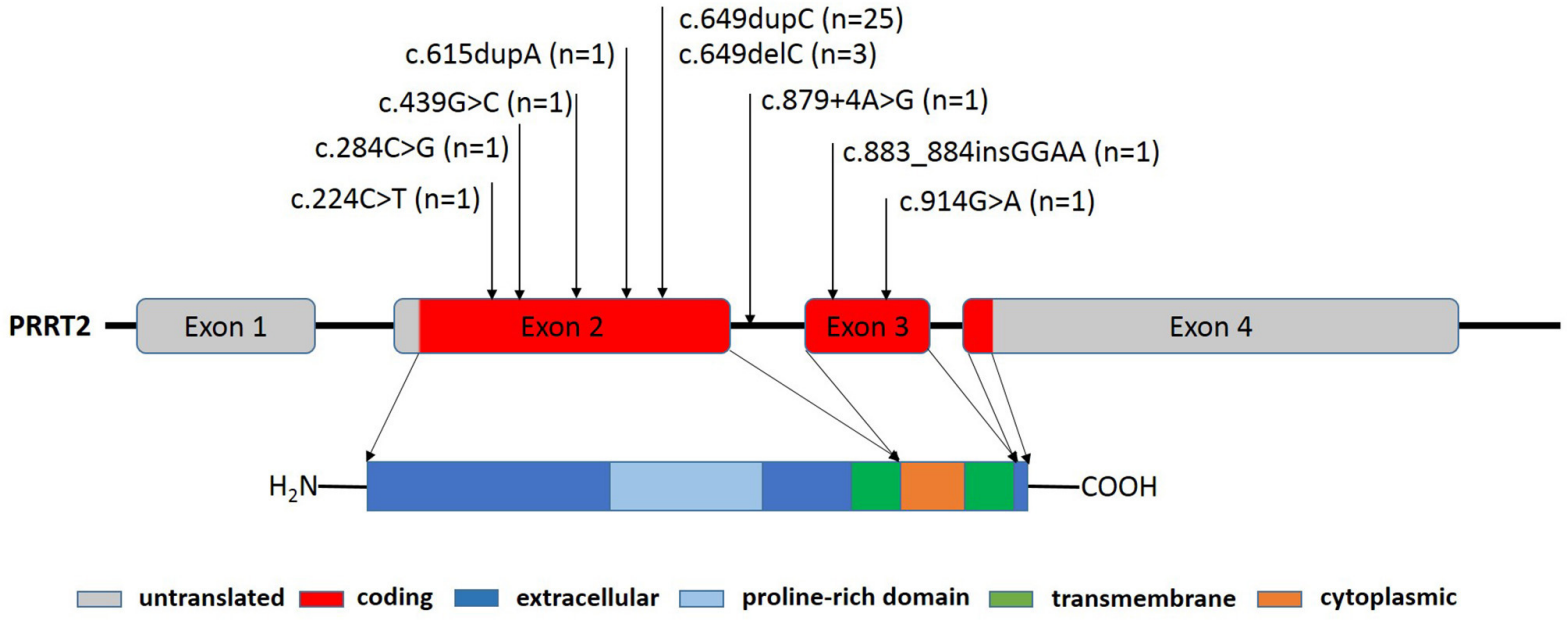
PRRT2 gene structure and locations of PRRT2 mutations in the PRRT2 gene sequence. The PRRT2 gene contains four exons that encode a 340 amino acid protein, including a protein-rich domain (amino acids 131-216) in its extracellular region (amino acids 1-268), one cytoplasmic region (amino acids 290-317), and two transmembrane regions (amino acids 269-289 and 318-338). The positions of the 35 heterozygous mutations identified in this study are shown.

Forty-four patients (29 males and 15 females) with pathogenic *PRRT2* variants were identified (Table 1). Nine patients (20.5%) showed *PRRT2* deletions (eight patients with *PRRT2* whole gene deletions, and one with 16p11.2 microdeletion). The remaining 35 patients (79.5%) were *PRRT2* heterozygous variants, including 30 frameshift mutations, three missense mutations, one nonsense mutation, and one splice site change. Of the 35 variants, 32 (91.4%) coded the proline-rich domain and extracellular domain, including 25 c.649dupC mutations, three c.649delC mutations, one c.224C>T mutation, one c.2484C>G mutation, one c.439G>C mutation, and one c.615dupA mutation (Figure 1). Based on the Human Gene Mutation Database (HGMD), four patients displayed novel variants (c.284C>G, c.883_884insGGAA, c.879+4A>G, and c.914G>A). The nonsense variant c.284C>G (p.S95X) resulted in a premature stop codon. The c.883_884insGGAA (p.N296Kfs^*^45) produced a frameshift variant with a premature stop codon 45 amino acids downstream. Both of them were classified as “pathogenetic” according to the ACMG criteria. The c.879+4A>G variant affected the splice site. According to the ACMG criteria, it was classified as “variant of unknown significance.” The missense variant c.914G>A (p.G305E) affected highly conserved residues of the cytoplasmic domain toward the C terminus of PRRT2, which was considered “variant of unknown significance” according to the ACMG criteria. All missense variants were predicted to be damaging to protein function by prediction tools used (Table 2). Thirty-four (97.1%) of the frameshift mutations were inherited, and only one c.649dupC mutation (2.9%) was *de novo*.

**Table 1:** Genotypes of 44 patients with pathogenic PRRT2 mutations.

**Patient**	**Position:chr16**	**Exon**	**Variant**	**Protein**	**Novel/reported**	**Parental derivation**
P1	29824599	2	c.224C>T	p.P75L	R	M
P2	29824659	2	c.284C>G	p.S95X	N	M
P3	29824814	2	c.439G>C	p.D147H	R	M
P4	29824990	2	c.615dupA	p.S208Ifs^*^17	R	F
P5	29825024	2	c.649dupC	p.R217Pfs^*^8	R	F
P6	29825024	2	c.649dupC	p.R217Pfs^*^8	R	F
P7	29825024	2	c.649dupC	p.R217Pfs^*^8	R	M
P8	29825024	2	c.649dupC	p.R217Pfs^*^8	R	M
P9	29825024	2	c.649dupC	p.R217Pfs^*^8	R	F
P10	29825024	2	c.649dupC	p.R217Pfs^*^8	R	F
P11	29825024	2	c.649dupC	p.R217Pfs^*^8	R	M
P12	29825024	2	c.649dupC	p.R217Pfs^*^8	R	F
P13	29825024	2	c.649dupC	p.R217Pfs^*^8	R	M
P14	29825024	2	c.649dupC	p.R217Pfs^*^8	R	M
P15	29825024	2	c.649dupC	p.R217Pfs^*^8	R	M
P16	29825024	2	c.649dupC	p.R217Pfs^*^8	R	M
P17	29825024	2	c.649dupC	p.R217Pfs^*^8	R	M
P18	29825024	2	c.649dupC	p.R217Pfs^*^8	R	M
P19	29825024	2	c.649dupC	p.R217Pfs^*^8	R	F
P20	29825024	2	c.649dupC	p.R217Pfs^*^8	R	M
P21	29825024	2	c.649dupC	p.R217Pfs^*^8	R	M
P22	29825024	2	c.649dupC	p.R217Pfs^*^8	R	M
P23	29825024	2	c.649dupC	p.R217Pfs^*^8	R	F
P24	29825024	2	c.649dupC	p.R217Pfs^*^8	R	F
P25	29825024	2	c.649dupC	p.R217Pfs^*^8	R	*De novo*
P26	29825024	2	c.649dupC	p.R217Pfs^*^8	R	F
P27	29825024	2	c.649dupC	p.R217Pfs^*^8	R	F
P28	29825024	2	c.649dupC	p.R217Pfs^*^8	R	M
P29	29825024	2	c.649dupC	p.R217Pfs^*^8	R	F
P30	29825024	2	c.649delC	p.R217Efs^*^12	R	M
P31	29825024	2	c.649delC	p.R217Efs^*^12	R	F
P32	29825024	2	c.649delC	p.R217Efs^*^12	R	F
P33	29825657	3	c.883_884insGGAA	p.N296Kfs^*^45	N	F
P34	29825258	Splicing	c.879+4A>G	-	N	F
P35	29825688	3	c.914G>A	p.G305E	N	M
P36	29802081-30199897	16p11.2 Microdeletion			R	F
P37	-	Whole gene deletion			R	NA^*^
P38	-	Whole gene deletion			R	NA^*^
P39	-	Whole gene deletion			R	NA^*^
P40	-	Whole gene deletion			R	NA^*^
P41	-	Whole gene deletion			R	NA^*^
P42	-	Whole gene deletion			R	NA^*^
P43	-	Whole gene deletion			R	NA^*^
P44	-	Whole gene deletion			R	NA^*^

* *The patient's parents did not accept gene sequencing. NA, not available; R, reported; N, Novel; M, mother; F, father*.

**Table 2:** Pathogenicity prediction of three missense mutations.

**Variant**	**SIFT**	**PolyPhen 2**	**Mutation Taster**	**FATHMM-MKL**
c.224C>T, p.P75L	damaging	Benign	disease causing	Deleterious
c.439G>C, p.D147H	damaging	Benign	polymorphism	Tolerated
c.914G>A, p.G305E	damaging	probably damaging	disease causing	Deleterious

### Clinical Findings

**Figure 2:**
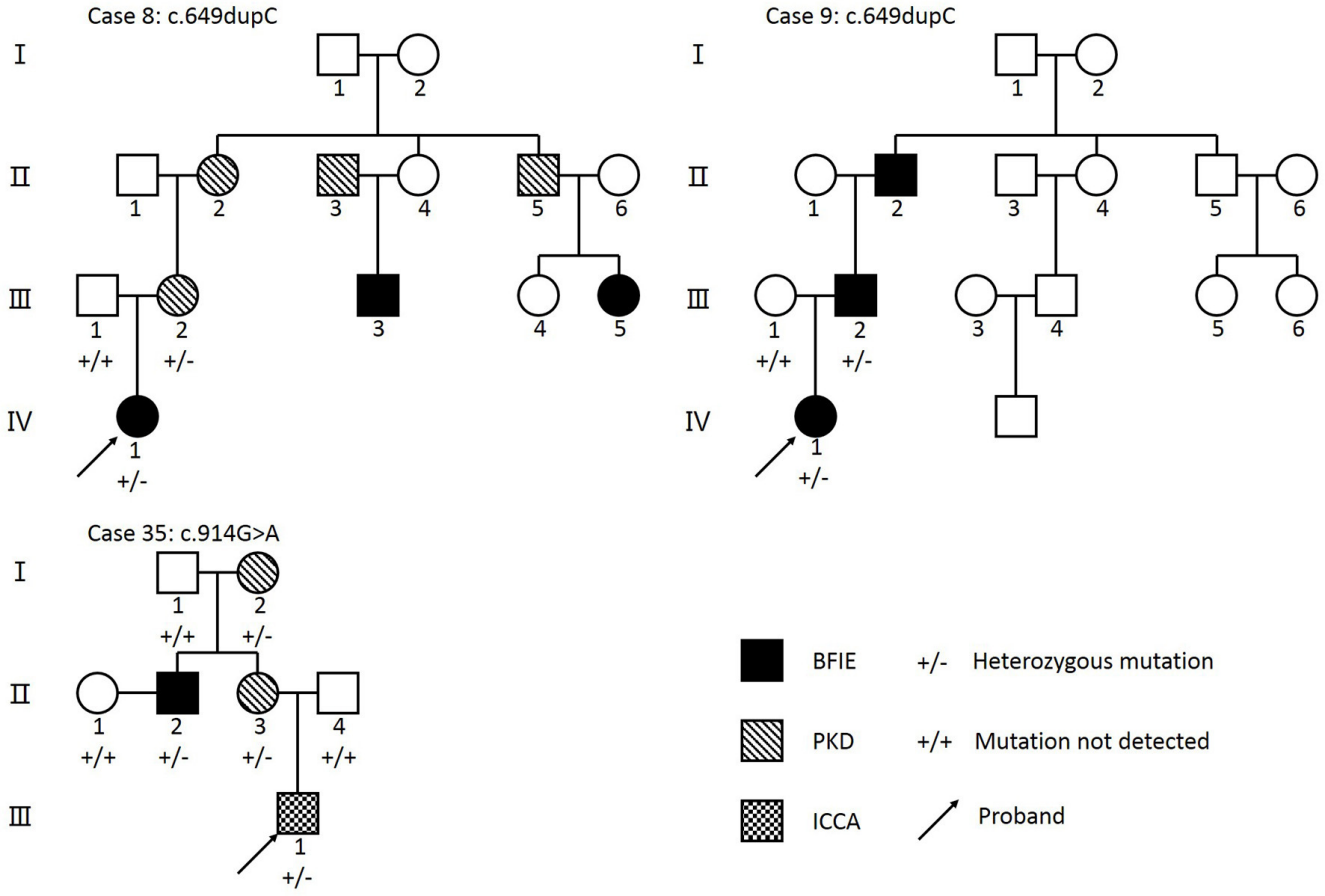
Pedigrees from three families with PRRT2 mutations. Arrow, proband; BFIE, benign familial infantile epilepsy; PKD, paroxysmal kinesigenic dyskinesia; ICCA, infantile convulsions with choreoathetosis. Individuals who underwent genetic sequencing are indicated by + and –. Individuals with a *PRRT2* heterozygous mutation are shown using +/–, and individuals that tested negative for a *PRRT2* mutation are shown using +/+. The c.649dupC mutation was identified in family 8 and family 9. The c.914G>A mutation was identified in Family 35.

Patients' detailed clinical information was gathered from 3 months to 2 years following *PRRT2* mutation diagnosis (Table 3). No specific perinatal history or abnormal physical examination were noted in any patients when admitted. Figure 2 presented three patients' pedigrees (Patients 8, 9, and 35) that showed segregation of *PRRT2* variants in more than two generations.

**Table 3:** Clinical characteristics of patients harboring pathogenic *PRRT2* mutations.

**Patient, Gender**	**Pheno-type**	**Variant**	**Family history^†^**	**Age of onset**	**Seizure Type**	**Ictal EEG**	**Age at treatment**	**Initial ASMs**	**Effective ASMs**	**Age at remission**	**Age at last follow up**	**Development**
P1, M	BFIE	c.224C>T	+/–	11 m	FBTC	NA	18 m	LEV	LEV	18 m	4 y	Normal
P2, M	BFIE	c.284C>G	+/–	4.5 m	FT	NA	5 m	LEV	LEV	5 m	4 y 3 m	Normal
P3, M	PKD	c.439G>C	+/–	13 y	Dystonia	NA	15 y	LTG	OXC	17 y	17 y	Normal
P4, M	BFIE	c.615dupA	–/–	5 m	FT, FBTC	NA	5.5 m	LEV	LEV+PB	Lost to follow up		
P5, M	BFIE	c.649dupC	–/+	3.5 m	FT	NA	4 m	LEV	OXC	4.5 m	4 y 9 m	Normal
P6, F	BFIE	c.649dupC	+/–	4.5 m	FBTC	NA	5 m	LEV	LEV	5 m	4 y 1 m	Normal
P7, F	BFIE	c.649dupC	+/–	3.5 m	FT, GTC	NA	4 m	LEV	LEV+TPM	12 m	3 y 8 m	Normal
P8, F	BFIE	c.649dupC	+/–	3.5 m	FBTC	NA	4 m	LEV	LEV	5 m	3 y 8 m	Normal
P9, F	BFIE	c.649dupC	+/–	3.5 m	FT	NA	4 m	LEV	OXC	5.5 m	3 y 7 m	Normal
P10, F	BFIE	c.649dupC	+/–	4 m	FT	NA	4.5 m	LEV	LEV	4.5 m	3 y 5 m	Normal
P11, F	BFIE	c.649dupC	–/–	5.5 m	FBTC	NA	7 m	LEV	LEV	7 m	3 y 6 m	Normal
P12, M	BFIE	c.649dupC	–/+	5 m	FT	NA	6 m	LEV	LEV	8 m	3 y 1 m	Normal
P13, F	BFIE	c.649dupC	–/–	4.5 m	FT, FBTC	NA	5 m	LEV	LEV	5.5 m	2 y 9 m	Normal
P14, M	BFIE	c.649dupC	+/–	4 m	FT	NA	4.5 m	LEV	LEV+VPA	8 m	2 y 9 m	Normal
P15, M	BFIE	c.649dupC	+/–	3.5 m	FBTC	Temporal	4 m	LEV	VPA	4.5 m	2 y 9 m	Autism
P16, F	BFIE	c.649dupC	+/–	3.5 m	FT	Temporal	4 m	VPA	VPA	4 m	2 y 9 m	Normal
P17, M	BFIE	c.649dupC	+/–	3.5 m	FT, GTC	Frontal	4 m	LEV	LEV	Lost to follow up		
P18, M	BFIE	c.649dupC	–/–	5.5 m	FT, GTC	NA	5.5 m	LEV	LEV+VPA	8 m	2 y 8 m	Normal
P19, F	BFIE	c.649dupC	+/–	4.5 m	FBTC	NA	5 m	LEV	LEV	6 m	2 y 7 m	Normal
P20, M	BFIE	c.649dupC	+/+	4 m	FT, GTC	Frontal	4.5 m	LEV	LEV	Lost to follow up		
P21, M	BFIE	c.649dupC	+/–	5.5 m	FBTC	Tem	6 m	OXC	OXC	6 m	1 y 8 m	Normal
P22, M	BFIE	c.649dupC	+/–	5 m	FT	NA	6 m	OXC	OXC	6 m	1 y 8 m	Normal
P23, F	BFIE	c.649dupC	+/–	4.5 m	FBTC	Frontal	5 m	LEV	OXC	5.5 m	7 m	Normal
P24, F	BFIE	c.649dupC	+/–	4 m	FT	NA	4.5 m	LEV	LEV+PB	5.5 m	4 y 5 m	Normal
P25, M	BFIE	c.649dupC	–/–	5 m	FT	NA	5.5 m	LEV	LEV	Lost to follow up		
P26, M	BFIE	c.649dupC	+/–	5 m	FBTC	NA	5.5 m	LEV	LEV	Lost to follow up		
P27, M	BFIE	c.649dupC	–/–	7.5 m	FT	NA	8 m	OXC	OXC	Lost to follow up		
P28, M	PKD	c.649dupC	–/+	9 y	Choreo-athetosis	NA	9.5 y	OXC	OXC	Lost to follow up		
P29, M	BFIE	c.649dupC	–/+	4.5 m	FT, GTC	Frontal	5.5 m	LEV	OXC	6 m	2 y 1 m	Normal
P30, M	BFIE	c.649delC	+/–	4.5 m	FT, GTC	Tempotal	5 m	LEV	OXC	11 m	1 y 6 m	Normal
P31, M	BFIE	c.649delC	+/–	6 m	FT	NA	6.5 m	LEV	OXC	7 m	1 y 6 m	Normal
P32, M	BFIE	c.649delC	+/–	4 m	FT	NA	4.5 m	VPA	VPA	6 m	4 y	Normal
P33, F	BFIE	c.883_884insGGAA	+/–	4.5 m	FBTC	NA	5 m	LEV	LEV	14 m	3 y 1 m	Normal
P34, M	BFIE	c.879+4A>G	+/+	3.5 m	FBTC	NA	4 m	OXC	OXC	Lost to follow up		
P35, M	ICCA	c.914G>A	–/+	6 y	Choreo-athetosis	NA	6 y	OXC	OXC	6.5 y	8 y	Normal
P36, M	ICCA	16p11.2 Microdeletion	–/–	10 y	Choreo-athetosis	NA	12.5 y	OXC	OXC	12.5 y	13 y 9 m	DDID
P37, M	BFIE	WGD	+/–	12 m	FBTC	NA	None	None	None	14 m	4 y 5 y	Normal
P38, F	BFIE	WGD	–/–	3.5 m	FBTC	NA	4 m	LEV	LEV	8 m	3 y 6 m	Normal
P39, M	BFIE	WGD	–/–	3 m	FBTC	NA	4 m	LEV	LEV+VPA	5 m	3 y 5 m	Normal
P40, F	BFIE	WGD	–/–	8.5 m	FT	NA	16 m	LEV	OXC	26 m	4 y	Normal
P41, F	BFIE	WGD	+/–	4 m	FT	Occipital	4.5 m	LEV	LEV+VPA	12 m	4 y 6 m	Normal
P42, M	BFIE	WGD	–/–	5 m	FT	NA	5 m	LEV	LEV	14 m	3 y	Normal
P43, M	BFIE	WGD	–/–	8.5 m	FT, GTC	NA	18 m	OXC	OXC	18 m	2 y 11 m	Normal
P44, M	BFIE	WGD	+/–	8 m	FBTC	NA	None	None	None	12 m	-	Death

† *Self-limited non-febrile seizures during infantile period/paroxysmal kinesigenic dyskinesia. EEG, electroencephalogram; ASMs, antiseizure medications; M, male; F, female; BFIE, benign familial infantile epilepsy; PKD, paroxysmal kinesigenic dyskinesia; ICCA, infantile convulsions with choreoathetosis; m, month; y, year; GTC, generalized tonic-clonic seizure; FBTC, focal to bilateral tonic-clonic seizure; FT, focal tonic seizure; LEV, levetiracetam; OXC, oxcarbazepine; VPA, valproic acid; LTG, lamotrigine; DDID, developmental delay/intellectual disability. WGD, whole gene deletion*.

Forty patients (25 males and 15 females) with pathogenic *PRRT2* variants were diagnosed with BFIE, and 29 of them had a family history of seizures or PKD. The average age of onset was 5.1 ± 2.0 months. There was no significant difference (4.7 vs. 6.6 months, *P* = 0.143) in age of onset between patients with heterozygous mutations (*n* = 32) and patients with whole gene deletions (*n* = 8). All the patients presented with focal motor seizures. Twenty-three patients had focal to bilateral tonic-clonic seizures. Twenty-eight patients presented seizures in clusters (2-20 attacks per day). Status epilepticus was noted in one patient (Patient 44). Ictal EEG showed that discharges originated from temporal lobe (Patients 15, 16, 21, and 30), frontal lobe (Patients 17, 20, 23, and 29) and occipital lobe (Patient 41).The interictal EEGs were normal in 35 patients, whereas 4 patients showed interictal spike waves that originated from the frontal lobe (Patients 38 and 42), temporal lobe (Patient 33), or occipital lobe (Patient 14) during frequent attacks, which disappeared during subsequent follow-up. All patients demonstrated a normal brain MRI.

Two patients were diagnosed with PKD (Patients 3 and 28). Both of them had a family history of movement disorders. The age of symptoms onset was 9 and 13 years old, respectively. Patient 3 presented with dystonia triggered by standing up suddenly, and patient 28 presented with bilateral choreoathetosis triggered by sudden movement. The attacks occurred several times per day and usually lasted <1 min without loss of consciousness. Both patients showed normal interictal EEG and brain MRI.

Two patients (Patients 35 and 36) were diagnosed with ICCA. Patient 35 had manifested self-limited clusters of non-febrile focal motor seizures since the age of 5 months and developed bilateral dyskinesia at the age of 6 years. Patient 36 had focal to bilateral tonic-clonic seizures from 8 to 18 months of age, and presented with unilateral choreoathetosis at 10 years old. These two ICCA patients had normal brain MRI and EEG.

### Treatment and Outcome

A total of 38 patients with BFIE initiated ASMs at the average age of 5.9 ± 3.5 months (Table 4). Thirty-one patients received levetiracetam (LEV; 10 mg/kg/day), and 16 (51.6%) patients were seizure free while using a dose between 15 and 40 mg/kg/d. However, the remaining 15 (48.4%) patients still experienced seizures after the LEV dosage was gradually increased to 30-40 mg/kg/d. Of these 15 patients, seven reached seizure freedom after the addition of phenobarbital (PB), topiramate (TPM), or valproic acid (VPA), respectively. Another seven patients were switched to oxcarbazepine (OXC; 10-20 mg/kg) and the seizures were completely controlled. The remaining one patient became seizure free after switching to VPA. Meanwhile, five patients were initially treated with OXC (10 mg/kg/d), and all of them reached seizure freedom at 15-20 mg/kg/d OXC. Two patients with BFIE initially received VPA (20 mg/kg/d), and their symptoms were controlled.

**Table 4:** Treatment of *PRRT2*-associated disorders.

	**BFIE (*n* = 40)**	**PKD/ICCA (*n* = 4)**
Age of onset	5.1 ± 2.0m	9.5 ± 2.9y
Age at treatment	5.9 ± 3.5m	10.8 ± 3.9y
Initial ASMs	LEV (77.5%) VPA (5.0%) OXC (12.5%) None (5.0%)	OXC (75.0%) LTG (25.0%)
Response to initial ASMs	LEV (51.6%) VPA (100%) OXC (100%)	OXC (100%) LTG (0%)
Effective ASMs	LEV (42.1%) VPA (7.9%) OXC (31.6%) Combination (18.4%)	OXC (100%)

*m, months; y, years; ASMs, antiseizure medications;*

*LEV, levetiracetam; OXC, oxcarbazepine; VPA, valproic acid;*

*LTG, lamotrigine*.

Two patients (Patient 37 and 44) with BFIE did not receive treatment due to their parents' personal decision. Patient 37 was seizure free 2 months after the first seizure. Patient 44 presented with focal to bilateral tonic-clonic seizures in clusters since the age of 8 months. Interictal EEG during hospitalization once showed bilateral centro-frontal epileptic activity. Unfortunately, he died of status epilepticus (duration unknown) at home without any emergency aids at the age of 12 months.

The average age of seizure remission in the 32 patients with BFIE that received follow-up was 8.5 ± 5.0 months. Compared with patients with heterozygous mutations (*n* = 25), patients with whole gene deletions (*n* = 7) had a significantly later remission (13.9 vs. 7.1 months, *P* = 0.001). All patients had normal developmental milestones during follow-up except Patient 15 who developed autism at 2 years old.

One patient with PKD and two patients with ICCA were initially administered OXC. Patient 28 was lost to follow up, and the remaining two patients remitted from dyskinesia at a dose of 10-20 mg/kg/d. Patient 3 with PKD was initially administered lamotrigine (LTG, dosage unavailable) with no response. The attacks were controlled after switching to OXC (10 mg/kg/day). The average age at last episode was 12 ± 5.3 years. One patient with 16p11.2 microdeletion (Patient 36) showed mild developmental delay/intellectual disability. The remaining patients had normal cognitive outcome.

## Discussion

*PRRT2* gene locates on chromosome 16p11.2, and encodes the proline-rich transmembrane protein 2 that is mainly expressed in the cerebral cortex, basal ganglia, and cerebellum (Figure 1). The function of the *PRRT2* gene remains unclear, but current findings indicate that it is involved in the process of Ca^2+^-related presynaptic neurotransmitter release (8). A recent study demonstrated that *PRRT2* also interacts with the voltage-gated Na^+^ channels Na_v_1.2 and Na_v_1.6 to negatively modulate neural activity (9). Thus, pathogenic mutations in the *PRRT2* gene may lead to a state of neuronal hyper-excitability, which clinically presents a series of paroxysmal disorders. In this study, we reported 44 children with *PRRT2* pathogenic mutations. In line with current data, the most common variant was the c.649dupC frameshift mutation, which was identified in 56.8% of patients in our study (4). We also reported four novel variants. The c.284C>G (p.S95X) and the c.883_884insGGAA (p.N296Kfs^*^45) led to a truncated *PRRT2* protein and were classified as “pathogenetic” according to the ACMG criteria. The splice site variant (c.879+4A>G) was inherited from patient's father with cosegregation consistence which suggested its pathogenicity, although it was classified as “variant of unknown significance” according to the ACMG criteria. But further studies are needed to confirm its pathogenicity. The missense variant c.914G>A (p.G305E) was predicted to be damaging by Mutation Taster, Polyphen2, SIFT and FATHMM-MKL (Table 2). According to the study from Tsai et al., pathogenic missense variants at the C terminus often lead to failure of protein targeting to the plasma membrane that could be an important mechanism for *PRRT2*-associated disorders (10).

Several studies have reported the existence of homozygous mutations, compound heterozygous mutations, and 16p11.2 microdeletion of *PRRT2* in a small group of cases (11–14). In 2015, Ebrahimi-Fakhari et al. summarized that these rare cases accounted for 1.5% (21/1,444) of all *PRRT2*-associated cases (4). Meanwhile, only 3.7% of patients were identified with a whole deletion of *PRRT2* gene in a recent Italian cohort study (15). In this study, the proportion (20.5%) of patients with whole gene deletions or 16p11.2 microdeletion was significantly higher than previous data. This could be explained by the selective bias due to different standards for accepting genetic testing in clinical practice. The biallelic mutation or complete deletion of the *PRRT2* gene led to total impairment of function of the *PRRT2* protein, possibly resulting in the patient's severe clinical presentation. In our cohort, one patient suffered status epilepticus, and one patient showed mild developmental delay/intellectual disability. Both of them had complete deletions of the *PRRT2* gene. In addition, we found that patients with whole gene deletions had a later remission in *PRRT2*-associated BFIE. Previous study have revealed that patients with homozygous, compound heterozygous and microdeletion mutation more frequently presented with more severe phenotypes including intellectual/development disorders when comparing with heterozygous mutations cases (16–18). However, evidences remain insufficient to prove the existence of genotype-phenotype correlations among *PRRT2*-associated disorders. The “gene-dosage effect” that causes severe impairment or total loss of the *PRRT2* protein that leads to clinical presentations still require further validation.

Yang et al. found that the whole *PRRT2* gene mutation and the 16p11.2 microdeletion were more likely be *de novo* (19)_._ Unfortunately, the parental derivation of eight patients with whole gene deletions was not available. Five of them had no family history, which could possibly serve as potential indirect evidence. However, seven family members carried a pathogenic mutation without clinical symptoms although *PRRT2*-associated disorders are autosomal dominant inherited, which suggested the phenomenon of incomplete penetrance. Balagura et al. reported that the penetrance of *PRRT2* pathogenic mutation was 89% (15). Schubert et al. estimated an 82% penetrance of *PRRT2* mutations in BFIE (20). Meanwhile, the penetrance of *PRRT2* mutations in PKD was estimated to be 61%, which was raised to nearly 90% when ICCA cases were taken into account (21).

All patients in this study were diagnosed with BFIE, PKD, or ICCA due to inclusion criteria, and their clinical characteristics were similar with previous studies. BFIE, PKD, and ICCA are main phenotypes of *PRRT2*-associated disorders, accounting for more than 90% of all cases (4). In addition, several studies have reported that other disorders, including hemiplegic migraine and episodic ataxia, could be a *PRRT2*-associated phenotype (4). In a recent study, migraine occurred as a concomitant diagnosis of BFIE in 10% of *PRRT2*-associated patients, suggesting an increased risk for migraine in younger patients with *PRRT2*-associated BFIE (22). *PRRT2-*associated disorders are relatively benign. However, recent studies suggested that pathogenic *PRRT2* mutation could be responsible for some severe epileptic syndromes. Döring et al. reported a case who presented BFIE at the age of 4 months and developed continuous spikes and waves during sleep at the age of 4 years (22). Only an inherited c.649dupC variant was identified through whole gene sequencing. Furthermore, Jafarpour and Desai reported that infantile spams could also be a *PRRT2*-associated phenotype (23). In addition, *PRRT2* mutations could be responsible for early childhood myoclonic epilepsy according to a recent research (18). These findings suggested that this evolving spectrum has a more widely boundary. However, further studies are needed to establish whether *PPRT2* mutations play an important role in these situations.

Symptoms of *PRRT2*-associated disorders are readily controlled by conventional ASMs (4). Monotherapy was effective in a majority of patients with BFIE in our study. Considering both the efficacy and safety, LEV is one of the first-line choices for clinical treatment of pediatric epilepsy, especially for infantile patients. Most patients in our study were initially treated with LEV. Experiences from Zhao et al. suggested that OXC seems to be more effective than LEV (24). Similarly, half of patients in our study had no response to LEV, whereas all patients treated with OXC quickly became seizure free. These results revealed that OXC seems to be superior to LEV for patients with *PRRT2*-associated BFIE. OXC is a derivative of carbamazepine (CBZ) that has fewer side effects and drug interactions (25). Pan et al. suggested that OXC had a significant effect on pediatric PKD (26). Moreover, compared with non-*PRRT2* mutations cases, CBZ seemed to be more effective in patients with *PRRT2*-associated PKD (27). A recent study revealed that *PRRT2* could serve as a negative modulator of sodium channels (9). Therefore, as sodium channel blockers, CBZ and OXC probably have specific mechanisms for controlling symptoms in *PRRT2*-associated disorders. In addition, Symonds et al. recently reported that *PRRT2*-associated BFIE was the most frequent single-gene epilepsy, with an incidence of 1 per 9,970 live births (28). Thus, early genetic testing for patients with suspected BFIE is helpful to clinical treatment. Nevertheless, considering the self-limited course of *PRRT2*-associated diseases, whether ASMs play an essential role is ambiguous. However, one patient without ASMs suffered status epilepticus and died of delayed treatment, suggesting that the appropriate treatment is still necessary. Thus, comprehensive evaluation should be individualized when planning the treatment regime for patients with *PRRT2*-associated disorders.

Outcome of patients with *PRRT2*-associated disorders is usually favorable. In the present study, the average age of remission of patients with BFIE was 8.5 ± 5.0 months, which was similar to previously published data (29). Most patients had normal psychomotor development at last follow-up. Interestingly, one patient carrying the c.649dupC variant showed language retardation that was diagnosed with autism after seizure remission. Although microdeletions and duplications at 16p11.2, where the *PRRT2* gene is located, have been observed in patients with autism (30, 31). Current studies have found no clear relationship between *PRRT2* pathogenic mutation and autism (32). Thus, we postulated that *PRRT2* mutations play no pathological role in the development of autism in this patient. However, the function of the *PRRT2* gene during the psychomotor development period needs further study.

This study has several limitations. Firstly, patients with BFIE may not yet develop movement disorders during the relatively short follow-up. Meanwhile, considering the self-limited course of *PRRT2*-associated disorders, some patients did not accept gene tests. Moreover, we did not include patients with other rare phenotypes. Thus, our results may not reflect the accurate phenotype distribution. Secondly, pedigree analysis was limited due to insufficient data of family members. Thus, continuous follow-up and detailed information of probands and family members are needed to better understand the characteristics of *PRRT2*-associated disorders.

In conclusion, pathogenic *PRRT2* mutations are responsible for a series of paroxysmal diseases, mainly including BFIE, PKD, and ICCA. Patients with whole gene deletions could have more severe phenotypes. Conventional antiseizure medications, especially OXC, could be the first-line choice. Further long-term cohort studies and pedigree analysis are needed to fully illuminate characteristics of *PRRT2*-associated diseases.

## Data Availability Statement

The original contributions presented in the study are included in the article/supplementary material, further inquiries can be directed to the corresponding author/s.

## Ethics Statement

The studies involving human participants were reviewed and approved by the ethics committee of Children's Hospital of Chongqing Medical University. Written informed consent to participate in this study was provided by the participants' legal guardian/next of kin.

## Author Contributions

LJ and L-lX designed the study. S-qH, X-jL, ML, YH, J-nM, PW, MZ, MC, and T-sL collected the patients' information. H-yL performed the statistical analysis. H-yL and L-lX drafted first version of the manuscript. All authors participated in the critical review of the manuscript, contributed to the article, and approved the submitted version.

## Conflict of Interest

The authors declare that the research was conducted in the absence of any commercial or financial relationships that could be construed as a potential conflict of interest.
